# The Socioeconomic and Institutional Determinants of Participation in India’s Health Insurance Scheme for the Poor

**DOI:** 10.1371/journal.pone.0066296

**Published:** 2013-06-21

**Authors:** Arindam Nandi, Ashvin Ashok, Ramanan Laxminarayan

**Affiliations:** 1 The Center for Disease Dynamics, Economics & Policy, Washington D.C., United States of America; 2 Public Health Foundation of India, New Delhi, India; London School of Economics, United Kingdom

## Abstract

The *Rashtriya Swasthya Bima Yojana* (RSBY), which was introduced in 2008 in India, is a social health insurance scheme that aims to improve healthcare access and provide financial risk protection to the poor. In this study, we analyse the determinants of participation and enrolment in the scheme at the level of districts. We used official data on RSBY enrolment, socioeconomic data from the District Level Household Survey 2007–2008, and additional state-level information on fiscal health, political affiliation, and quality of governance. Results from multivariate probit and OLS analyses suggest that political and institutional factors are among the strongest determinants explaining the variation in participation and enrolment in RSBY. In particular, districts in state governments that are politically affiliated with the opposition or neutral parties at the centre are more likely to participate in RSBY, and have higher levels of enrolment. Districts in states with a lower quality of governance, a pre-existing state-level health insurance scheme, or with a lower level of fiscal deficit as compared to GDP, are significantly less likely to participate, or have lower enrolment rates. Among socioeconomic factors, we find some evidence of weak or imprecise targeting. Districts with a higher share of socioeconomically backward castes are less likely to participate, and their enrolment rates are also lower. Finally, districts with more non-poor households may be more likely to participate, although with lower enrolment rates.

## Introduction

Despite rapid economic growth that has lifted millions out of poverty in recent decades; the slow pace of improvement in the health of India’s population has generated a fierce debate on India’s health policy [1]. Three-quarters of healthcare spending in India, or $14.5 billion in 2005 [2], is borne out of pocket [3]. Out of pocket health spending is a major cause of impoverishment, pushing approximately 3.5% to 6.2% of the population below the poverty line every year [2], [4], [5], [6], [7].

The appropriate balance between supply- and demand-side initiatives to improve health outcomes is still a subject of considerable debate. India has historically focused on supply-side health policies, from the Rural Health Policy (1977) and its grassroots-level community health workers, to the comprehensive National Rural Health Mission (NRHM) of 2005 that provided additional resources for healthcare delivery in poorer states. Recently, a report of the Planning Commission’s High Level Expert Group (HLEG) on universal health coverage in India [8] has recommended a supply-side programme that would assimilate the current piecemeal supply- and demand-side policies into a streamlined National Health Package (NHP). It envisions a system where every citizen would have full access to free healthcare from either a public healthcare provider or a private provider working under a government contract. The proposed system would resemble the National Health Service in the United Kingdom, with primary, secondary, and tertiary care financed by the national government but provided by a combination of governmental and non-governmental agencies.

However, during the past decade, the government has experimented with demand-side policies that complement service delivery. Following the success of the *Janani Suraksha Yojana* (Safe Motherhood Scheme), a conditional cash transfer program for pregnant mothers [9], the *Rashtriya Swasthya Bima Yojana* (RSBY, National Health Insurance Program) was introduced in 2008. The main goal of RSBY is to encourage access to healthcare, especially in areas where public health facilities are either unavailable, of poor quality, or overburdened.

While public health facilities are cheaper (in terms of out-of-pocket expenditure) and physically near many villages, poor quality of care, high rates of absenteeism, shortage of equipment contributes to the lack of healthcare access. These shortcomings have made private practitioners the dominant treatment providers to the poor, especially in rural areas. For example, an ongoing mapping study (Medical Advice Quality and Availability in Rural India, or MAQARI) finds that more than 92% of rural households visit private providers, and 79% visit providers with no formal medical training [10]. RSBY’s approach is to insure the poor against health shocks by providing them with access to both public healthcare, and more expensive private healthcare, which they could not otherwise afford. Thus, it will serve the dual purpose of improving healthcare access while providing financial protection to the poor [11], [12]. In fact, some also argue that the incentives embedded in the RSBY scheme can themselves address some of the system’s deficits by inducing private healthcare supply in poor and rural markets [13].

There is an ongoing debate about the merits of RSBY. For example, RSBY only covers inpatient services, (which are typically conducted at tertiary or secondary healthcare facilities) but does not cover primary or outpatient care [14]. However, pilot studies on outpatient coverage under RSBY are underway in two districts of Orissa and Gujarat (according to RSBY Connect newsletter, February 2012, Government of India). In addition, there are concerns about RSBY’s administrative functionality and economic impact. Seshadri et al. (2012), from a survey of poor households in Gujarat, found that while hospitalization and utilization rates among RSBY beneficiaries were much higher compared to non-beneficiaries, there was no significant difference in the out-of-pocket medical expenditure between the two groups [15]. There were also serious administrative inefficiencies, e.g. in households who were already enrolled under the scheme, 30% of individual members were not enlisted, making those persons ineligible for benefits. Furthermore, due to concerns about the reimbursement process, many healthcare providers had reduced or stopped offering non-surgical procedures. Another study, Rajasekhar et al. (2011) found similar problems in Karnataka, such as almost zero RSBY utilization rates, partly due to lack of information among beneficiaries, but also due to refusal by care providers [16]. Various other studies have evaluated the economic impact and the long term financial viability of RSBY [17]–[20]. On the other hand, among similar schemes at the state level, a significant reduction in out-of-pocket medical expenditure among health insurance beneficiaries in Andhra Pradesh has been found [21].

Although RSBY’s overall success in enrolment is widely acknowledged [14], [22], there remain widespread disparities in enrolment. In this study, we analyse the factors contributing to variations in both participation and enrolment in RSBY across districts.

Although RSBY is mandated by the central government, decisions to participate in the scheme, and the rate of enrolment, may depend on various local socioeconomic, political, and institutional factors. For example, as the central and state governments (75% central funding, and 25% state funding, except for Jammu and Kashmir and North-eastern states where the ratio is 90% central funds and 10% state funds) jointly finance RSBY, the significant outlay expected of state governments may influence their decision to participate. Also, enrolment in RSBY is based on a list of officially poor households (below poverty level or BPL), which is prepared at the district level. Therefore, district level factors could affect uptake rates. Furthermore, since there are no mandates on the implementation of the scheme in the state (e.g. no definite timeline to complete enrolment), understanding the nature of participation and enrolment can shed some light on both the administrative and political bottlenecks, and the household level determinants of RSBY enrolment. Inequality in participation and enrolment can also help identify segments not reached by the program, and focus policymakers’ attention on prioritizing these segments. The yet uncovered population can be targeted with enhanced enrolment campaigns, and any barriers to entry for additional private providers can be removed.

Using RSBY administrative data on enrolment, matched district-level data from the District Level Household Survey 2007–2008, and data on state fiscal health, political affiliation and quality of governance, we examine the socioeconomic and institutional determinants of the participation and enrolment in RSBY. We find that political and institutional factors are among the strongest determinants of both participation and enrolment. In particular, we find that states with pre-existing health insurance schemes for the poor, and counter-intuitively, states in better fiscal health are less likely to participate in RSBY.

Since centre-state politics may play an important role, we examine factors such as the political affiliation of the ruling party, and control for the quality of governance. We find that these factors significantly affect both participation and enrolment rates. Surprisingly, districts located in states that are neutral, or closely affiliated to the opposition parties at the centre, are significantly more likely to implement RSBY, and yield a higher enrolment rate, versus districts in states ruled by parties that are part of the ruling United Progressive Alliance (UPA) coalition at the centre. The effect is stronger for districts in states that are part of the opposition at the centre. Finally, we find some correlation between socioeconomic household specific factors (such as religion, wealth, and gender of household head) and RSBY participation and enrolment.

### Description of the RSBY Program

With a coverage of more than 171 million people at present, an annual budget of $96 million (assuming US$ 1 = Rs. 50) in 2009–10 [14], [23], and a final target enrolment of 300 million by 2013, RSBY is among the world’s largest health insurance programs. Originally, households below the official poverty line (BPL) were eligible to participate in RSBY. However, the government has recently decided to include additional non-BPL but socioeconomically disadvantaged groups under RSBY, such as domestic workers, or workers in construction or *beedi* industries [24].

RSBY has a complex structure, with the involvement of several stakeholders, and requiring intensive monitoring by the state government. It is implemented by the state nodal agency, an independent body established to implement the scheme in the state. The nodal agency is typically the Department of Labour (in almost half of the states), but may also be under the Ministry of Health [25]. The scheme has pioneered a public-private partnership approach, with state governments contracting out the provision of insurance coverage to private insurance companies. In exchange for a registration fee (Rs. 30 or US$0.6) paid to the government, enrolled households are entitled to receive inpatient healthcare coverage up to Rs. 30,000 or US$600 per year (for a family of 5). The scheme also provides money for transport charges at the rate of Rs. 100 (US$2) per visit, with an annual maximum of Rs. 1000 (US$20). Households must renew their enrolment in the scheme every year.

The central and state governments pay the remainder of the premium for each enrolled family directly to the insurance companies. Premium rates are determined at the level of a district, through a tendering process among insurance providers by the state government. The state government also empanels various public and private healthcare providers under the scheme. Financial transactions between a covered household and healthcare providers are “cashless”, and payment is made on the basis of a biometric RSBY Smart-card. Insurance coverage is only applicable for inpatient care, and a beneficiary can purchase these treatments and procedures at a government-fixed price from the provider. Payment is made using the biometric card, i.e. the insurance provider reimburses the treatment cost directly to the healthcare provider.

The enrolment process begins with the state government providing the insurer with an electronic list of eligible BPL households. This list is prepared at the district level. In close cooperation with a district level officer, the insurer then publicizes the enrolment schedule and a registration location. All members of an eligible family must visit the enrolment centre, and are enrolled in the presence of a government officer and an insurance company representative. The list of households that have been enrolled is then collected by the nodal agency. Later, it is maintained centrally, and is the basis for financial transfers from the central government to the state governments (at a 75∶25 ratio, as mentioned earlier). The scheme also provides for the inclusion of intermediaries such as NGOs to provide grassroots outreach and assist members in utilizing the services after enrolment [26], [27].

### Existing Literature on RSBY Enrolment

The current literature on RSBY enrolment is rather limited. While some studies have examined the enrolment patterns at national and state levels, analyses at more disaggregated levels of governance are relatively scarce. Furthermore, research examining RSBY enrolment patterns have been mostly descriptive. In this section, we will briefly discuss a few of these studies, along with the findings related to uptake rates of similar community insurance programs in other countries.

A working paper examining 24 districts across seven Indian states, does not find any evidence of gender, age, or demographic bias among RSBY’s enrolled population [28]. Based on village level probit analysis, the author concludes that villages have a higher probability of participation if there are more eligible families, and better access to commercial facilities. The study also shows that remote villages have lower enrolment rates. The results, although limited by low explanatory power of the model, also exhibit a positive association between the local capacity to provide public health services and enrolment rates. The study speculates on the roles of poor-quality BPL lists, inadequate logistical planning and preparation, and selective enrolment (based on cost of enrolment) by insurance companies in determining enrolment rates, but does not provide any empirical evidence.

Using a larger sample of 145 districts during the first year of RSBY, Swarup (2011) finds district-wise imbalances in enrolment rates [29]. The descriptive analysis by the author largely attributes these variations in enrolment rates to “defective and outdated” BPL lists provided by the state governments to insurance companies, and concludes that the errors in the lists also produce a gender bias, since they include only the names of male heads of household. However, this could also be attributed to skewed incentives for insurance companies because payment is provided on enrolment per household instead of enrolment per individual [30].

Dror and Vellakkal (2012) evaluate the financial burden of RSBY, and its implications for enrolment [22]. The authors argue that finance plays an important role in undertaking enrolment drives. In order to scale up from the existing levels of enrolment, and to maintain the financial viability of the scheme, the central budget allocation for RSBY should be increased and the scheme will have to attract a large above-poverty-line enrolment (i.e., those who pay a non-subsidized premium).

Studies on community health insurance programs in other countries reveal that the evidence on enrolment equity across socioeconomic groups is mixed [31], [32]. Jehu-Appiah et al. (2011) conducts a household level analysis of the Ghana National Health Insurance Scheme to determine that enrolment is influenced by socioeconomic factors, health status, and social perceptions [33]. Basaza et al. (2007) argue that low enrolment under the Ugandan Health Insurance Scheme is a result of limited community involvement and outreach, a lack of trust in the management of the scheme, and people's inability to pay the premiums [34]. While the design and operation of RSBY differs from community health insurance schemes abroad, some of the analytical results point to similar factors associated with enrolment and participation gaps.

In summary, the literature on RSBY points to the importance of three factors in raising enrolment rates: strengthening information and educational campaigns [30], [35], strengthening administrative capabilities that lead to updated BPL lists and the involvement of local administrators [28], [29], and improving financial outlays [22].

## Methods

### 1. Data and Descriptive Statistics

Our data come from two main sources. RSBY enrolment data which are continually updated are obtained from the official RSBY web portal [36]. District-level enrolment rate (ranging from 0 to 1) is calculated from these data as a ratio of the total number of enrolled BPL families to the total number of target BPL families. At the time of data collection, RSBY was either not rolled out, or enrolment data were not available for all districts. For this study, 384 districts from 22 states and a union territory have a non-zero enrolment rate. Zero-enrolment-rate districts exist in situations where RSBY is either completely absent from the entire state containing that district (e.g. Andhra Pradesh), or where RSBY is being implemented in other districts of the state (e.g. Rajasthan).

**Figure 1:**
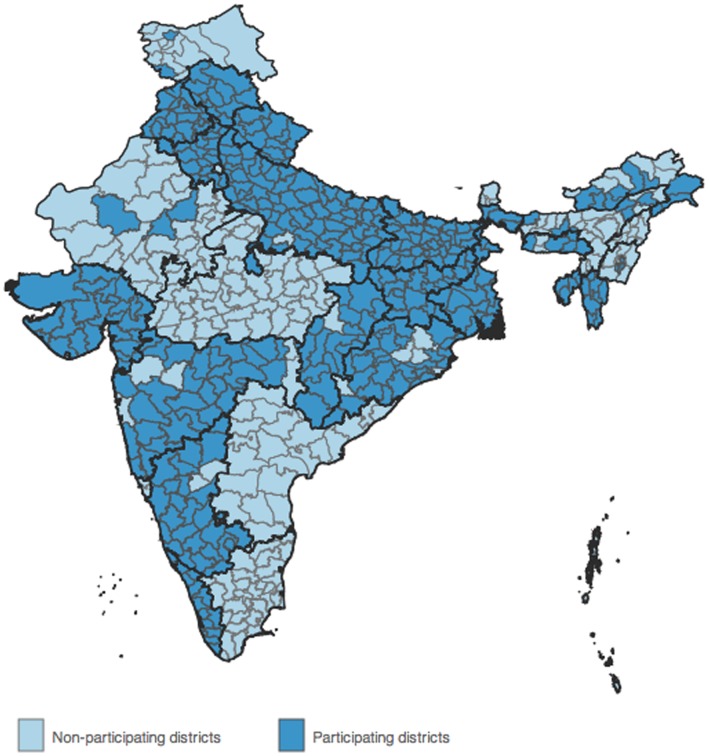
RSBY participating districts. Source: RSBY website (www.rsby.gov.in) downloaded on 22 May 2012. Map created using GADM data. www.gadm.org.

**Figure 2:**
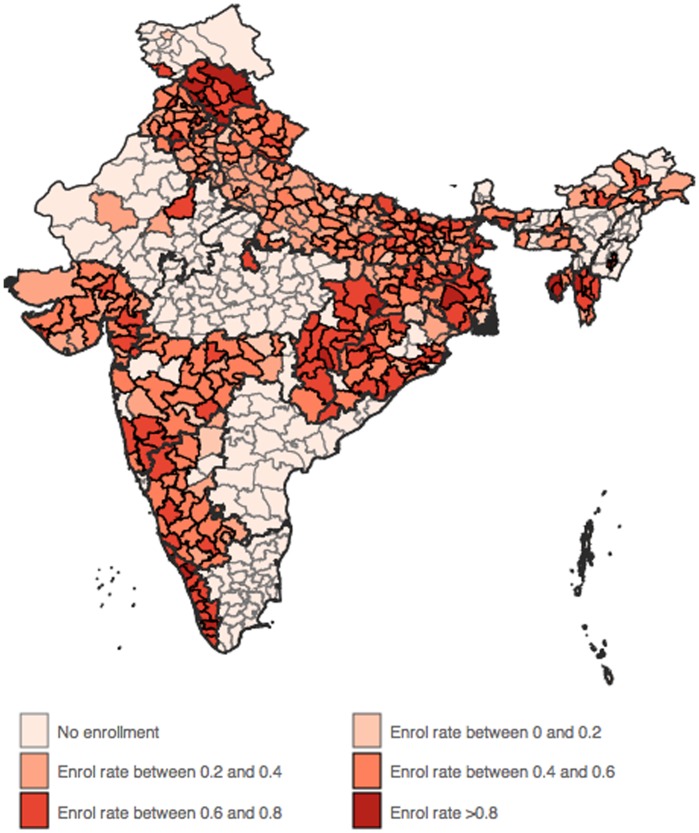
Variation in RSBY enrolment rates. Source: RSBY website (www.rsby.gov.in) downloaded on 22 May 2012. Map created using GADM data. www.gadm.org.

Figures 1 and 2 illustrate this variation in participation and enrolment rates across districts in India. Table 1 also presents the state wise number of districts for which these administrative data were available, along with the median RSBY enrolment rate. The commencement date of the scheme in each district is also available in the data, and is used to control for enrolment time in our analysis, i.e. the number of days that the scheme has been in effect in a particular district (from commencement to May, 2012).

**Table 1:** Number of RSBY participating districts by state.

State	Non-participating districts	RSBY Participating districts (with data)	Total no. of districts in state	Median enrolment rate
Andhra Pradesh	23		23	0.000
Arunachal Pradesh	6	10	16	0.442
Assam	22	5	27	0.531
Bihar		37	37	0.550
Chhattisgarh	1	15	16	0.657
Goa	2		2	0.000
Gujarat		25	25	0.507
Haryana		20	20	0.416
Himachal Pradesh		12	12	0.821
Jammu & Kashmir	12	2	14	0.401
Jharkhand		22	22	0.489
Karnataka	2	25	27	0.514
Kerala		14	14	0.749
Madhya Pradesh	45		45	0.000
Maharashtra	7	28	35	0.518
Manipur	6	3	9	0.686
Meghalaya	2	5	7	0.434
Mizoram		8	8	0.676
Orissa	5	25	30	0.601
Punjab		20	20	0.501
Rajasthan	29	3	32	0.373
Sikkim	4		4	0.000
Tamil Nadu	30		30	0.000
Tripura		4	4	0.724
Uttar Pradesh	1	69	70	0.366
Uttarakhand		13	13	0.519
West Bengal	1	18	19	0.635
Andaman & Nicobar	2		2	0.000
Chandigarh		1	1	0.508
Dadra & Nagar Haveli	1		1	0.000
Daman & Diu	2		2	0.000
Delhi	9		9	0.000
Lakshadweep	1		1	0.000
Pondicherry	4		4	0.000
Total	217	384	601	0.515

Source: http://rsby.gov.in/, data downloaded on 22 May 2012. Note: Total no. of districts in states reflect those surveyed in DLHS 2007–08. Since the survey, additional districts have been created, but are not mentioned in this table. Districts in Nagaland have not been included since no DLHS data are available for the state.

We matched data from the RSBY web portal with data from the District Level Household Survey (DLHS) 2007–2008 [37], a large cross-sectional survey of more than 600,000 Indian households, except for the state of Nagaland. DLHS collected household-level information on various socioeconomic indicators such as caste, religion, location (rural or urban) and ownership of several types of assets. Due to the large sample size, estimates from DLHS are representative at the district level. We use district level socioeconomic characteristics of households, the economic status of villages in terms of availability of roads, electricity, and public health facilities and the administrative capacity at the local level measured by the average number of schemes implemented per village in a district as the determinants of RSBY enrolment in our analysis.

Since a state’s participation in RSBY will likely depend on political and institutional factors, we use additional data on the state’s fiscal health as measured by the ratio of gross fiscal deficit to state GDP (From Reserve Bank of India data [38]), institutional environment or quality of governance as measured by a corruption index (based on a mix of perception and experience surveys [39]), and state political affiliation vis-a-vis the central government.

Table 2 presents summary statistics of our compiled dataset. In total, 590 districts are included in our analysis, of which 384 districts across 22 states and one union territory (Chandigarh) have a non-zero enrolment rate. Of the 601 districts in DLHS, 9 districts were omitted due to absence of village level infrastructure information, and 2 districts in Andaman and Nicobar islands were omitted because the corruption index data were not available.

**Table 2:** Descriptive Statistics- Means, Standard deviations and number of observations for model variables.

Variables	Non-participating districts (enrolment dummy = 0)			Participating districts (enrolment dummy = 1)			Total sample		
	Mean	Std. dev.	No. of districts	Mean	Std. dev.	No. of districts	Mean	Std. dev.	No. of districts
Enrolment rate	0.000	0.000	217	0.516	0.173	384	0.329	0.284	601
**Household level variables**									
*Share of households that are/have:*									
Rural	0.725	0.248	217	0.788	0.160	384	0.765	0.199	601
Female Household head	0.102	0.064	217	0.121	0.068	384	0.114	0.067	601
Scheduled Caste	0.164	0.090	217	0.190	0.094	384	0.181	0.093	601
Scheduled Tribe	0.212	0.268	217	0.160	0.251	384	0.178	0.258	601
Other Backward Caste	0.393	0.219	217	0.364	0.210	384	0.375	0.214	601
Hindu	0.776	0.275	217	0.771	0.246	384	0.773	0.257	601
Muslim	0.118	0.202	217	0.098	0.119	384	0.105	0.154	601
Christian	0.067	0.176	217	0.055	0.170	384	0.059	0.172	601
Sikh	0.005	0.018	217	0.040	0.148	384	0.027	0.120	601
Buddhist	0.018	0.095	217	0.016	0.070	384	0.016	0.080	601
*Share of households that belong to:*									
Wealth quintile 1	0.176	0.179	217	0.198	0.197	384	0.190	0.191	601
Wealth quintile 2	0.176	0.119	217	0.198	0.120	384	0.190	0.120	601
Wealth quintile 3	0.208	0.096	217	0.191	0.077	384	0.197	0.085	601
Wealth quintile 4	0.208	0.102	217	0.206	0.114	384	0.207	0.110	601
Wealth quintile 5	0.232	0.206	217	0.207	0.175	384	0.216	0.187	601
**District level variables**									
*Share of villages in a district that have:*									
All weather roads	0.848	0.150	208	0.870	0.139	384	0.862	0.143	592
Electricity	0.903	0.143	208	0.830	0.227	384	0.855	0.204	592
Public Health facilities	0.507	0.227	208	0.494	0.234	384	0.498	0.232	592
Average no. of schemes implemented per village in district	6.989	2.268	208	7.661	2.216	384	7.425	2.256	592
Enrolment time (days)**	0	0	217	334	177	384	213	214	601
**State level variables**									
Gross Fiscal Debt to State GDP ratio	2.875	1.619	215	3.106	1.595	384	3.023	1.606	599
Corruption index	2.893	1.108	215	2.352	1.211	384	2.546	1.202	599
Ruling party	0.585	0.494	217	0.339	0.474	384	0.428	0.495	601
Opposition party	0.392	0.489	217	0.471	0.500	384	0.443	0.497	601
Neutral party	0.023	0.150	217	0.190	0.393	384	0.130	0.336	601
Any political change between 2008 and 2012†	0.166	0.373	217	0.367	0.483	384	0.295	0.456	601
Degree of political shift††	−0.300	0.859	217	0.258	1.042	384	0.057	1.015	601
Other insurance schemes	0.263	0.441	217	0.102	0.302	384	0.160	0.367	601

* Average no. of schemes implemented assesses the implementation of all schemes surveyed in DLHS 2007–08 at the village level.

** Enrolment time measures the number of days between commencement of enrolment in district and May 22, 2012.

† Any political change since 2008 is a binary variable capturing any change in ruling party since 2008.

†† Degree of political shift is an interaction term capturing swing in political affiliation (based on affiliation index) given political change since 2008.

*** Data sources: District level data from DLHS 2007–08, Enrolment rate information from http://www.rsby.gov.in, State fiscal data from Reserve Bank of India (2013)- State Finances A Study of Budgets of 2012–13 and Corruption index constructed from TII-CMS 2007 survey.

### 2. Empirical Approach

Three different sets of models are estimated to determine the factors affecting RSBY participation and enrolment. First, we estimate a multivariate probit model of the binary indicator of the RSBY participation (i.e. non-zero enrolment) for all 590 districts. This is followed by an OLS model of RSBY enrolment rate, which includes both zero and non-zero enrolment districts. Finally, we examine the determinants of enrolment rate only among the 384 districts that participate in RSBY (i.e. non-zero enrolment).

Since RSBY has not yet been adopted in all states and districts (i.e., some states and districts have zero enrolment), we tested for self-selection bias on the OLS estimates using tests for endogeneity. The results show very weak and non-robust evidence of self-selection. The absence of any strong self-selection bias could be a result of using districts as the unit of analysis, instead of using households or individuals as units of analyses.

We include a range of socioeconomic, political, and institutional factors as explanatory variables in our probit and OLS models. From DLHS data, we include the district level share of rural households, households with a female head, various castes (scheduled castes, scheduled tribes, and other backward castes), and religions (Muslim, Christian, Sikh, and Buddhists, as compared with Hindus, the largest religious group) that could impact participation and enrolment. To assess the effect of local infrastructure, also included are district level shares of villages with all-weather roads, electricity, and at least one public health facility.

To capture the effect of standard of living of the households in a district, we first use a principal component analysis to create a household asset index from DLHS (following Filmer and Pritchett 2001 [40]). The household variables that are used to create the asset index are the possession of tangible assets, such as radio, sewing machine, TV, bicycle, car, and others, along with indicators of housing condition, such as construction quality, availability of toilets, sources of drinking water, and type of cooking fuel. Households in the entire sample are divided into five quintiles or standard of living groups based on this asset index. The shares of households that belong to quintiles 2 to 5 in a district are then included among the explanatory variables.

Given the significant state budget required to implement RSBY, a state’s fiscal health could play an important role in determining the extent of its participation [22].We use the ratio of gross fiscal deficit to gross state GDP, obtained from the Reserve Bank of India data [37], as a possible determinant in our analysis. RBI (2013) Table IV.3 provides the state level average GFD/GSDP ratios for the years 2004–2008, 2010–2011 and 2011–2012. We took an overall average over this period to derive the GFD/GSDP ratios. For UTs not included in this table, we used the average central government’s fiscal deficit to GDP ratio from www.indiastat.com).

Anecdotal evidence suggests that the political affiliation of the state government, relative to the party in power at the national level, may be an important determinant of a state’s willingness to participate in RSBY [41]. To capture this relationship, we construct a state-level political affiliation index, which measures a state’s political proximity to the ruling UPA coalition at the centre.

The affiliation index reflects the political identity of the state government as of May 2012, and has the following values: 0 = BJP (*Bharatiya Janata Party*, the principal opposition party at centre); 1 = National Democratic Alliance (NDA) coalition partner; 2 = Indian National Congress (INC) is principal opposition party/UPA coalition partner is principal opposition party at state; 3 = Neutral party; 4 = UPA coalition partner; 5 = ruling party at centre, INC. NDA is a coalition of opposition parties at the center, including BJP and seven other smaller political parties. Neutral parties are not affiliated with either the NDA coalition or the UPA coalition. Broadly speaking, the closer the state government is to the national government, the higher the value of the index.

From this index, two binary indicators for affiliation (for the *i-th* state) to the ruling party or neutral party are constructed and included in the right hand side of the regression:
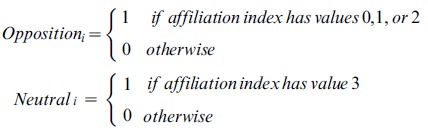



The inclusion of the two binary variables allows us to measure the effect of a state’s proximity to opposition parties at the centre, or its neutrality with respect to the UPA coalition at the centre.

While political affiliation reflects the relationship of state ruling parties with the centre as of May 2012 (i.e. at the time of our RSBY data collection), it is also important to incorporate any changes in the affiliation (as a result of election) since the conception of RSBY in 2008. To this end, we include a binary variable for change as a covariate. Additionally, in the event that a political change did occur, the swing in political affiliation is captured by another variable measuring the difference in ‘before’ and ‘after’ political affiliations. For example, Goa was ruled by the Congress from 2007 to 2012, and then went to the BJP. Based upon the coding of our political affiliation index, Goa’s political swing = –5. Table 3 presents an overview of the political affiliation of Indian states.

**Table 3:** State level political and corruption classifications.

States	Ruling Party	Affiliation	Affinity level	Pol change between 2008–2012	Political Swing	Corruption Index
Andhra Pradesh	INC	UPA	5	0	0	1
Arunachal Pradesh	INC	UPA	5	0	0	2
Assam	INC	UPA	5	0	0	4
Bihar	JD(U) BJP	NDA	1	0	0	4
Chhattisgarh	BJP	NDA	0	0	0	2
Goa	BJP	NDA	0	1	−5	4
Gujarat	BJP	NDA	0	0	0	2
Haryana	INC	UPA	5	0	0	1
Himachal Pradesh	BJP	NDA	0	0	0	1
Jammu & Kashmir	JKNC	UPA	4	0	0	4
Jharkhand	BJP	NDA	0	1	0	2
Karnataka	BJP	NDA	0	0	0	3
Kerala	INC	UPA	5	1	5	2
Madhya Pradesh	BJP	NDA	0	0	0	4
Maharashtra	INC	UPA	5	0	0	1
Manipur	INC	UPA	5	0	0	2
Meghalaya	INC	UPA	5	1	2	3
Mizoram	INC	UPA	5	0	2	1
Orissa	BJD	–	2	0	0	2
Punjab	SAD	NDA	1	0	0	1
Rajasthan	INC	UPA	5	0	0	3
Sikkim	SDF	–	3	0	0	3
Tamil Nadu	AIADMK	–	2	1	−2	3
Tripura	CPI (M)	–	2	0	0	1
Uttar Pradesh	SP	–	3	1	0	4
Uttarakhand	INC	UPA	5	1	5	1
West Bengal	TMC	UPA	4	1	2	1
Andaman & Nicobar	UPA	UPA	5	0	0	Not Available
Chandigarh	UPA	UPA	5	0	0	1
Dadra & Nagar Haveli	UPA	UPA	5	0	0	1
Daman & Diu	UPA	UPA	5	0	0	1
Lakshadweep	UPA	UPA	5	0	0	2
Delhi	INC	UPA	5	0	0	1
Pondicherry	INRC	UPA	5	0	0	1

**Source:** Political affiliation data compiled from various sources. Corruption Index is from TII-CMS 2007. Affiliation index coded as 0 = BJP (principal opposition party at centre); 1 = National Democratic Alliance (NDA) coalition partner; 2 = Indian National Congress (INC) is principal opposition party/UPA coalition partner is principal opposition party, at state; 3 = Neutral party; 4 = UPA coalition partner; 5 = ruling party at centre, INC.

The administrative capacity of a state’s bureaucracy may also significantly influence its participation in RSBY. This is particularly due to the complex structure of RSBY, as discussed in section 2. We try to capture this in our covariates in two ways. First, DLHS collects data on the number of government schemes (from a list of schemes) already implemented in each village. We compute the average number of schemes implemented by villages in each district, and use it as an indicator of the bureaucratic efficiency of a district.

The level of corruption is another possible proxy for the quality of governance. We use the Transparency International India–Centre for Media Studies India (TII-CMS) corruption index as a second measure for the quality of governance. These data are publicly available at the state level from the report of the TII-CMS India Corruption Survey 2007. The survey groups states into four categories of the level of corruption: moderate, high, very high, and alarming.

This corruption index is suitable for our analysis for two reasons. First, the TII-CMS survey measures both corruption perception and experience across 11 public services in various sectors (including the public distribution system, hospitals, electricity, water, and the National Rural Employment Guarantee Scheme). Second, the survey measures not only perception, but also the experience of BPL households in dealing with the government.

A state-level index for various levels of corruption (for the *i-th* state) as below, has also been included among explanatory variables:
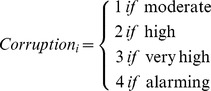



Finally, a state’s willingness to participate in the central RSBY scheme may depend on whether the state has already implemented, or is in the process of implementing, its own social health insurance program. From various sources such as state agency websites, we collected data on these state level schemes. We found five states with such schemes: Andhra Pradesh (*Rajiv Aarogyasri* Scheme), Goa (Mediclaim Scheme), Karnataka (*Vajpayee Arogyasri* Scheme/*Yesashwini*), Kerala (Comprehensive Health Insurance Scheme), and Tamil Nadu (Chief Minister *Kalaignar* Insurance Scheme). In Kerala, the Comprehensive Health Insurance Scheme covers the BPL families who are not covered by the RSBY. Also, the insurance is available to non-BPL families at a non-subsidized premium.
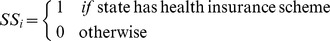



Among other state health insurance schemes, Maharashtra rolled out its *Rajiv Gandhi Jeevandayee Arogya Yojana* in July 2012, and Rajasthan has recently announced *Mukhyamantri* BPL *Jeevan Raksha Kosh*. Since our enrolment data are dated May 2012, we did not consider Maharashtra or Rajasthan in our analysis. An indicator of such state-level schemes, for the *i-th* state as below, is included in our covariates:

The OLS models of enrolment rate include an additional explanatory variable – a measure of enrolment time (in days), which is the difference between the commencement date and the date of our RSBY data collection. The OLS regressions are first performed on all districts in the sample, and then on a restricted sample of participating districts.

## Results and Discussion

### 1. Probit Regression of RSBY Participation

Table 4 presents the results from the probit and OLS regression models. Results from the probit model show that some socioeconomic characteristics of districts have a significant effect on a district’s participation in RSBY. We observe that the districts with more female-headed households have a greater probability of participating in RSBY. This can be explained by a positive association between female leadership at household or community level, and access to public goods in India, as other studies have pointed out. For example, Jalan and Ravallion (2003) [42] find that female-headed households have greater access to piped water, while Bhalotra and Clots-Figueras (2011) [43] show that state assembly constituencies with women politicians are more likely to have public health facilities, and better antenatal care and immunization rates. Chattopadhyay and Duflo (2004) [44], and Andersen et al. (2008) [45] also discuss the implications of female leadership.

**Table 4:** RSBY Participation and Enrolment rate regression estimates.

	Probit(Marginal Effect)	OLS(All districts)	OLS(Participating districts)
	Model (I)Participation	Model (II)Enrolment rate	Model (III)Enrolment rate
*Share of households that are/have:*			
Rural	−0.343	−0.000	0.139
Female Household head	0.925**	0.550***	0.216
Scheduled Castes	−0.511	−0.268*	−0.058
Scheduled Tribes	−0.410*	−0.210**	−0.118
Other Backward Castes	−0.356**	−0.126*	−0.073
Muslim	−0.071	0.098	0.151*
Christian	−0.396**	0.166*	0.183**
Sikh	1.481**	−0.220***	−0.232***
Buddhist	−0.131	−0.027	−0.105
*Share of households that belong to:*			
Wealth Quintile 2	0.695**	0.168	−0.120
Wealth Quintile 3	0.113	−0.418***	−0.527***
Wealth Quintile 4	0.636*	0.265*	0.160
Wealth Quintile 5	−0.108	−0.225*	−0.264*
*Share of villages per district that have:*			
All weather roads	0.123	0.153**	0.098
Electricity	−0.624***	−0.103*	0.043
Public health facilities	−0.004	−0.001	−0.034
Average no. of schemes implemented	0.007	−0.002	−0.002
Enrolment time (days)		0.001***	0.000
Fiscal Health (Gross Fiscal Deficit/GSDP)	0.045**	0.005	0.012*
Corruption Index	−0.208***	−0.103***	−0.061***
Opposition Party	0.435***	0.247***	0.117***
Neutral Party	0.210***	0.186***	0.125**
Any political change (2008–2012)	0.178***	−0.073***	−0.121***
Political swing	0.293***	0.045***	0.034***
State Insurance Scheme	−0.234**	−0.042	0.056
Constant		0.404***	0.510***
Psuedo *R^2^*/*R^2^*	0.531	0.619	0.262
Adjusted *R^2^*		0.603	0.210
Number of districts	590	590	384

Source: RSBY website (www.rsby.gov.in) for enrolment data, downloaded on 22 May 2012. DLHS 2007–2008 for socioeconomic data, RBI (2013) for state fiscal data, and corruption index is from CII-TMS (2007). Coefficients that are statistically significant at 10%, 5%, and 1% level are marked with *, **, and *** respectively. Huber-White robust standard errors are used in all regressions.

The importance of caste and religion as determinants of access to public goods in India has been previously shown [46]–[49]. For example, similar to the findings in Betancourt and Gleason (2000) [46], we observe that districts with a higher share of socioeconomically backward groups such as Scheduled Tribes (ST) and Other Backward Castes (OBC) are less likely to participate in RSBY. Given the stated pro-poor targeting of the RSBY, this is a very important finding, which shows that there is a clear gap between the policy and its practice. We also find that districts with a higher share of Christian households are less likely to participate, and districts with a higher share of Sikhs are more likely to participate in the program.

The correlation between standard of living variables and RSBY enrolment is not monotonic. Districts with more households in second and fourth wealth quintiles are more likely to participate in RSBY, compared to the poorest wealth quintile. This may indicate a so called ‘elite capture’, whereby the richer districts, by virtue of administrative and political clout, successfully draws resources from the program [49], [50]. Alternatively, a median district may be more efficient in implementing RSBY, as opposed to districts with a higher share of BPL households (i.e. those in the lowest quintile) who may not have the resources to implement RSBY, while districts with a large number of households in in quintile 5 may be too rich to merit participation.

The inconsistent targeting, as shown by our results, is a very important finding. Various studies have noted the poor quality of BPL lists in the context of RSBY, e.g. the lists are often outdated, or include non-eligible households while excluding eligible ones [29], [30]. While this cannot be directly tested, our results provide an indirect way to evaluating the pro-poor targeting. If the BPL lists were consistent, one would expect a consistently negative effect of our wealth status variables, with the effect size growing over quintiles.

Districts with a higher share of villages that are electrified are less likely to participate in the scheme, but the availability of all-weather roads or public health facilities do not have any significant impact. Sun (2010) [28] refers to the initial effect of power cuts in delaying RSBY enrolment, and how providers adapt by equipping themselves with power backup and generators. The negative association between electricity and enrolment seen in our results may indicate pro-poor targeting, whereby the scheme is first rolled out among resource-constrained villages. Furthermore, administrative capacity, as measured by the average number of schemes already being implemented in villages, has no significant effect on the likelihood of a district participating in the program. Another possible factor in the efficacy of implementation could be the nodal agency in the state. We find some additional evidence (not presented in the results), that the Department of Labour is associated with higher enrolment rates versus other nodal agencies. However, there does not seem to be any effect on the likelihood of participation.

Analysing the effect of state finances on participation, we find that districts in states with a higher ratio of gross fiscal deficit to gross state GDP are more likely to participate in the scheme. This may be due to two of reasons. First, states with poorer fiscal health may have a higher share of BPL population, and therefore may attract more resources. This residual effect may exist even after controlling for standard of living (wealth quintiles), since there are inconsistencies in the BPL lists. Secondly, a higher fiscal deficit may already reflect a predisposition to implementing expensive welfare measures. Both of these will make such a state more likely to implement RSBY. Since the fiscal deficit may not accurately reflect the quality of state expenditure, we also measured the relationship between Non Development Revenue Expenditure (NDRE) of the state and participation, to conclude that states with greater levels of NDRE are less likely to participate in the scheme.

Among the political variables, we find that districts located in states that are ruled by parties closely affiliated with the ruling UPA coalition at the centre are significantly less likely to participate in RSBY. There is currently no analytical evidence for the effect of political affiliation on RSBY participation, although this relationship has been previously mentioned in media outlets [41]. In fact, the union labour minister of India recently claimed that three major Congress ruled states, Andhra Pradesh, Rajasthan, and Maharashtra, were making inadequate efforts to implement RSBY [51]. Analysing his comments further, we note that all three states have pre-existing, or planned, state level health insurance schemes. Our results show that such states are less likely to participate in RSBY, as discussed later in this section. Therefore, Congress rule by itself may not explain the negative association between UPA-allegiance and RSBY participation. There may be additional political factors, at a more disaggregated level, which are systematically different between the UPA-affiliated states and others. Sun (2010) [28], discusses the importance of lower level politics and the role of leaders in attracting enrolment camps to villages with a greater proportion of BPL families. The author implies that villages with a larger number of BPL families may be able to attract enrolment camps due to strong political ties between the village administration and higher levels of authority. Unfortunately, due to data paucity, the analysis of such factors is beyond the scope of this study.

Our results indicate that there may not be any political stigma associated with implementing a scheme championed by another political party. Even controlling for political changes in the interim period 2008–2012, i.e. after the official commencement of RSBY, we find that the political affiliation results are robust. Furthermore, districts in states, which witness a political change during 2008–2012, are more likely to participate in the scheme. Finally, we see that given a political change, districts in states that shifted closer towards the ruling coalition are also more likely to take up the program.

We find a negative association between higher levels of corruption, as measured by the corruption index, and RSBY participation. It echoes the current empirical evidence on the ill effects of corruption on the provision of public goods [52], [53], [54]. Over and above the conventional explanation of a lack of capacity to deliver a program, the negative association between corruption and participation may also reflect a lack of confidence among households in the administrative capability of the local government. Therefore, the perceived utility of participating in the program will be low, as seen in the case of some other schemes around the world [33].

Note however, a possible counter argument is that states with higher level of corruption may be more inclined to implement a scheme such as RSBY. Given the very high level of central funding (at least 75%), RSBY may provide an opportunity for further corruption. Therefore, the negative effect of corruption, as seen in our results, may be an underestimation. Furthermore, although the corruption measure used in this paper - a mix of perception and experience would be better that an index solely based on perception, systematic differences in self-reporting across population subgroups cannot be captured in our study.

We also find that states with their own pre-existing health insurance programs are significantly less likely to participate in RSBY. As mentioned in [14],) the five states with their own health insurance schemes for the poor have already budgeted substantial sums of money (e.g. the *Rajiv Aarogyasri* scheme in Andhra Pradesh allocated $21.5 million in 2009–10), and in some cases have more ambitious goals (covering more ailments and population segments). It is therefore not a surprise that these states are less likely to participate in RSBY.

### 2. OLS Regression of RSBY Enrolment Rate

The last two columns in Table 4 present OLS estimates of RSBY enrolment rates. The first set of estimates is based on a sample of all districts, and results in the last column are from a sample of participating districts only. We observe that similar to the probit model, factors such as caste and gender of the household head are statistically significant determinants of enrolment rates. In particular, districts with a higher share of SC, ST, or OBCs have a lower enrolment rate in model (II), although none of these caste variables (or household head’s gender) are significant in model (III). We also find some significant effect of religion.

In contrast with estimates for participation, the wealth quintile coefficients in the enrolment regression models indicate stronger pro-poor patterns. We see that districts with a high share of households in wealth quintiles 3 and 5 are associated with lower rates of enrolment in comparison with households in quintile 1.

Infrastructure in villages plays a significant role in explaining enrolment rates in model (II). Districts with a greater share of villages connected by all-weather roads are associated with higher rates of enrolment, while districts with a larger proportion of villages with electricity have lower enrolment rates. However, conditional on participation, these factors do not have any significant effect on enrolment rates, as seen in model (III).

The association between state fiscal health and enrolment appears to be weak or insignificant, but political and institutional factors remain very strong determinants of enrolment rates. Similar to model (I), corruption index is negatively associated with higher enrolment rates, and districts in ruling party states appear to have lower enrolment rates in comparison to others.

However, we find a negative effect of political change in the period 2008–2012 on enrolment rates in both models. When seen together with the results in model (I), this coefficient implies that while a change in the state government is associated with a higher likelihood of participating in RSBY (regardless of the affiliation of the new government), the political change may be detrimental to lower level bureaucracy, which controls the RSBY enrolment. There may be a lag in the information flow between the state government and administration at lower levels, controlling for time elapsed after enrolment commences in a district, which may thwart enrolment activities.

The coefficients of political swing imply that given a state undergoes a political change; higher enrolment rates are associated with states affiliated with the ruling UPA at the centre. Finally, pre-existing state insurance schemes have no statistically significant impact on the enrolment rates, and the time elapsed since the first implementation of RSBY in a district has some positive effect on enrolment in model (II).

It is important to note some limitations of our study. For example, we assume that the central government always provides full support for RSBY, and it is the state government that may choose to not roll out the program. However, as Dror and Vellakkal (2012) [22] point out, the budget allocation of the central government for RSBY falls well short of the projected expenditure, and the centre would find it difficult to adequately fund the scheme if all state governments were to fully embrace it. The authors also argue that premium rates are artificially low, often leading to a lack of enthusiasm from insurance providers. And, there may also be a discontent among the healthcare providers, as the government-fixed uniform prices for services are very low for some areas.

Secondly, due to lack of data, we cannot incorporate the role of some demand side factors in our study. In particular, as Das and Leino (2011) [30] point out, informational campaigns may have an important role to play in raising the awareness about RSBY. We assume that since the RSBY nodal agency and the state government are in charge of these campaigns, resources spent for such campaigns will be captured by other state level variables (e.g. fiscal health and standard of living) in our analysis.

Thirdly, although our results related to standard of living and socioeconomically backward groups show imprecise targeting, they are not a clear indicator of the problem related to BPL lists [28], [29]. Due to lack of data, we cannot directly evaluate the issue with these lists.

Finally, while the enrolment data are from 2012, the DLHS data are from 2007–08. This implies that there may be time varying factors, which may affect our results. Even though we include a measure of time elapsed since RSBY inception, as an explanatory variable, any large changes in other covariates, although unlikely, remain unobserved.

### Conclusion

The success of RSBY in achieving high enrolment rates is impressive, in comparison to much lower enrolment rates typically seen in other developing country [55], [56]. Our paper, which accounts for major socioeconomic, political, and institutional factors, points to some important conclusions. First, we find inequities in participation and enrolment by caste and religious groups. In particular, districts with socially backward communities (scheduled castes and scheduled tribes) experience lower participation and enrolment rates, and we also find some possible evidence of benefits being captured by higher wealth groups. These results reveal the weak nature of the pro-poor targeting mechanism of RSBY. Using these findings as a benchmark, the RSBY nodal agencies and state governments should strengthen the enrolment system, to align the resources with the needs of the people.

Secondly, institutional and political factors seem to be strongly correlated with both participation and enrolment rates. Districts in states with weaker administrative capacity, or those with pre-existing state health insurance program, are less likely to take up the RSBY program. Counterintuitively, opposition and non-UPA-allied states seem to be embracing the program far more enthusiastically than UPA-ruled states. The reasons for the lack of enthusiasm, though, are not clear (barring some speculative statements in newspaper articles) and merit further research.

Also, the impact of RSBY on improving population health, or reducing financial impoverishment, remains to be seen. The program faces significant challenges in its operating environment towards that objective. Supply-side problems include severe resource shortages (manpower, sanitation infrastructure, hospital beds, quality medicines), or physical distance from facilities. On the other hand, demand side issues such as costs or lack of awareness may result in underutilization of health services, especially in poor and marginalized communities [57].

Finally, there is a need for more disaggregated enrolment and claims data, which could offer useful insights about the time trends of participation, enrolment and utilization. While there is some research on the major determinants of RSBY utilization [12], more research is required to justify a large scale program such as RSBY. As Fan and Mahal (2011) [58] argue, there is a dire need for structured evaluation of health policies in India, a process that engages various stakeholders such as governments and the researcher community, facilitates flow of knowledge between the involved groups, and encourages evidence-based policymaking.
