# Correction: The Socioeconomic and Institutional Determinants of Participation in India’s Health Insurance Scheme for the Poor

**DOI:** 10.1371/annotation/c55e3dcd-dd24-47f7-9937-b1b7c5af7c5c

**Published:** 2014-01-06

**Authors:** Arindam Nandi, Ashvin Ashok, Ramanan Laxminarayan

A funding organization was incorrectly omitted from the Funding Statement. The Funding Statement should read: "Funding support by the Bill and Melinda Gates Foundation (Disease Control Priorities 3 project). The employers of the authors had no role in study design, data collection and analysis, decision to publish, or preparation of the manuscript." 

